# Cannabis use and suicide: a case-control study based on integrative data analysis

**DOI:** 10.1186/s42238-025-00352-1

**Published:** 2025-11-19

**Authors:** Qiongyu Shi, Guohua Li

**Affiliations:** 1 Department of Biostatistics, Columbia University Mailman School of Public Health, 722 West 168th St, New York, NY 10032 USA; 2 Department of Anesthesiology, Columbia University Vagelos College of Physicians and Surgeons, 622 West 168th St, New York, NY 10032 USA; 3 Department of Epidemiology, Columbia University Mailman School of Public Health, 722 West 168th St, New York, NY 10032 USA

**Keywords:** Cannabis, Case-control, Data fusion, Integrative data analysis, Multiple imputation, Suicide.

## Abstract

**Background:**

Cannabis use has been identified as a risk factor for depression, suicidal ideation, and suicide attempts. However, the direct relationship between cannabis use and suicide death has not been adequately investigated due to data and methodological challenges. We assessed the association between cannabis use and suicide using the case-control design and integrative data analysis techniques.

**Methods:**

Cases consisted of suicide decedents aged 16 years and older, selected from the 2013 National Violent Death Reporting System (NVDRS). Controls were respondents from the 2013 National Survey on Drug Use and Health (NSDUH). To mitigate potential misclassification in the NSDUH respondents’ self-reported cannabis use, we employed a data fusion approach by integrating the NSDUH data with data from the 2013 National Roadside Survey of Alcohol and Drug Use by Drivers (NRS), which included both toxicological testing and self-reported data on cannabis use. For statistical analysis, adjusted odds ratios (aORs) and 95% confidence intervals (CIs) of suicide associated with cannabis and alcohol use were estimated using weighted multivariable logistic regression models.

**Results:**

Multiply imputed toxicological testing data indicated that 17.1% of the cases, and 7.2% of the NSDUH controls used cannabis as indicated by testing positive for delta-9-tetrahydrocannabinol. Weighted multivariable logistic modeling revealed that cannabis use was associated with 83% increased odds of suicide (aOR = 1.83; 95% CI: 1.36, 2.31) and that alcohol use was associated with 20-fold increased odds of suicide (aOR = 20.53; 95% CI: 11.83, 29.24). Other factors associated with significantly increased odds of suicide were male sex, White race, being 35–49 years of age, and having less than a high school education.

**Conclusions:**

Cannabis use is a significant risk factor for suicide, independent of alcohol use and demographic characteristics. Policy makers should take into consideration the excess risk of suicide associated with cannabis use when assessing the externalities of legalizing cannabis.

## Background

Suicide is a major source of mortality worldwide. Each year over 720,000 individuals die by suicide globally (WHO 2025). In the United States, suicide rates have increased by 37% over the past two decades, claiming 49,476 lives in 2022 (CDC 2025). Use of alcohol and drugs has been recognized as an important risk factor for suicidality (Wong et al. 2013; Artenie et al. 2015; Poorolajal et al. 2016; Borges et al. 2017; Lange et al. 2024). Specifically, cannabis use as a contributing factor has been implicated in suicidal ideation and attempts among young adults, military veterans, and individuals with certain psychological conditions (Borges et al. 2016; Gobbi et al. 2019; Han et al. 2021; Daneshmend et al. 2022; Shamabadi et al. 2023). With more states legalizing cannabis for medical and recreational use, the prevalence of cannabis use among US adults aged 18 and older continues to rise (Hasin and Walsh 2021; Yang et al. 2023). Toxicological testing data indicate that the prevalence of cannabinoids detected in suicide fatalities increased from 8% in 2006 to 23% in 2017 (National Center for Drug Abuse Statistics 2025).

Previous studies assessing the role of cannabis use in suicidality relied on small sample sizes and self-reported data, limiting the validity and reliability of their findings. A major barrier to epidemiologic research on cannabis use and suicide has been the lack of toxicological testing data from representative samples of the general population. Although many studies examined the association between chronic cannabis use and suicidality, few have investigated the relationship between acute cannabis use and completed suicide. To assess the direct causal relationship between cannabis use and suicide in the US general population, we conducted a case-control study using data from multiple nationally representative samples.

## Methods

This research does not meet the definition of human subjects research under 45 CFR 46.

### Data sources

#### National Violent Death Reporting System (NVDRS)

The NVDRS is a comprehensive surveillance system of deaths from suicide and homicide in the United States. Each death record includes victim information, circumstances surrounding the death, and toxicological testing results wherever available (NCIPC 2022). In 2013, there were 17 states (Alaska, Colorado, Georgia, Kentucky, Maryland, Massachusetts, North Carolina, New Jersey, New Mexico, Ohio, Oklahoma, Oregon, Rhode Island, South Carolina, Utah, Virginia, and Wisconsin) participating in the NVDRS, representing approximately 30% of the US population (Lyons et al. 2016). In this study, we used the 2013 NVDRS as the data source of cases, operationally defined as individuals who died from suicide at age 16 years and older. Data on cannabis use and alcohol use for cases were based on toxicological testing results recorded in the NVDRS.

#### National Roadside Survey of Alcohol and Drug Use by Drivers (NRS)

The NRS is a field survey designed to monitor the prevalence of alcohol and drug use in the US driver population, which is sponsored by the National Highway Traffic Safety Administration and conducted every 7 to 10 years since 1973 (Kelley-Baker et al. 2016). The 2013 NRS is the most recently available data. In the 2013 NRS, drivers were randomly selected at 300 locations from 60 sites across the contiguous United States, and data for verbally consented drivers were collected during a single 2-hour Friday daytime session (either 9:30–11:30 AM or 1:30–3:30 PM) and four 2-hour nighttime periods (10:00 PM–midnight and 1:00 AM–3:00 AM on Fridays and Saturdays). Data collected included self-reported demographic information, breath samples, oral fluid samples, and blood samples. Survey data were weighted according to the multistage sampling scheme and the response rate (71.0%) to obtain unbiased estimates (Kelley-Baker et al. 2016). Data on self-reported quantity and frequency of cannabis use and alcohol use in the past year were included. In addition to self-reported cannabis and alcohol use, toxicological testing data were collected in the 2013 NRS.

#### National Survey on Drug Use and Health (NSDUH)

The NSDUH is an annual survey that collects comprehensive information about illicit drug use, alcohol consumption, and tobacco use among the US civilian, noninstitutionalized population aged 12 years and older. The 2013 NSDUH used a state-based sampling plan and a combination of computer-assisted personal interviewing and audio-computer-assisted self-interviewing techniques, with a response rate of 71.7%. The self-reported survey data were weighted to obtain unbiased national estimates for survey outcomes in the population represented by the 2013 NSDUH (SAMHSA 2014). The NSDUH data included respondent demographic characteristics, mental health conditions, and self-reported cannabis use and alcohol use in the past month. In this study, the 2013 NSDUH was used as the data source of substantiative controls with multiply imputed toxicological testing data on cannabis use and alcohol use through the algorithms developed based on the 2013 NRS dataset.

### Outcome measurements

The binary outcome measure was case-control status. Cases (*n* = 12,750) were individuals who died from suicide at age 16 years and older and who were recorded in the 2013 NVDRS, and controls (*n* = 43,465) were participants aged 16 years and older in the 2013 NSDUH.

### Exposure measurements

In the NVDRS, cannabis use was defined as a binary exposure, with a blood delta 9-tetrahydrocannabinol (THC) level above the detection limit (0.01 µg/mL) of the tests indicating positive. Alcohol use was also defined as a binary exposure, with a blood alcohol concentration (BAC) level ≥ 0.01 g/dL indicating positive.

In the NRS, cannabis use and alcohol use were measured in both blood and oral fluid samples. Similarly, cannabis use positivity was defined as testing positive for THC. Alcohol positivity was determined when a BAC level was ≥ 0.01 g/dL. Data on self-reported last-time cannabis use (past 24 h, past 2 days, past month, over a month, beyond a year/never) and the frequency of weekly alcohol use (0, 1–2, 3–4, 5–7, 8–14, more than 14 drinks) were also collected in the NRS. We converted the categorical self-reported data on cannabis use and alcohol use to be comparable with the self-reported past-month cannabis use and alcohol use in the NSDUH.

Because NRS participants were not a representative sample of the general population, it would be inappropriate to use them as controls. However, the NRS dataset contained both self-reported and toxicological testing data on cannabis and alcohol use, which allowed us to model the relationship between self-reported cannabis and alcohol use and toxicological testing results. In order to obtain toxicological testing data for the NSDUH respondents, we improvised integrative data analysis techniques by fusing toxicological testing data in the NRS into the NSDUH. Specifically, we developed prediction models using machine learning (Lasso logistic regression) to estimate toxicology-confirmed cannabis and alcohol use from self-reported data and demographic characteristics. These prediction models allowed us to combine the strengths of two datasets – NRS (with toxicological testing data) and NSDUH (with representative controls) – to generate toxicological testing data for appropriate controls to study the relationship between cannabis exposure and suicide. In addition, to address concerns related to missing data in the NRS, NVDRS, and the combined NRS-NSDUH dataset, we used multiple imputation techniques to create 20 imputed datasets with the Chained Equations Multiple Imputation algorithm. This data fusion with multiple imputation approach has been applied to assessing the association between cannabis use and homicide (Lee et al. 2024) and described in detail elsewhere (Yu et al. 2024).

### Covariates

Covariates included age (16–20, 21–34, 35–49, 50–64, and ≥65 years), sex (male vs. female), race (non-Hispanic White, non-Hispanic Black, Hispanic, and Others), and education (less than high school, high school graduate, some college, and college graduate or more). There were slight differences in categorizing age and race between datasets. We adopted the NSDUH categories by re-arranging some individuals into each category using raw data. For race/ethnicity, we re-grouped participants as Hispanic, non-Hispanic White, non-Hispanic Black, and all other non-Hispanic individuals as ‘Others’. We included these covariates because they are included in all three datasets using similar questionnaires, providing sufficient information for correct imputation. Additionally, they are well-established risk factors for suicide and potential confounders for the causal relationship between cannabis use and suicide.

### Statistical analysis

We first generated descriptive statistics to assess the distributions of the exposure variables and covariates. Then, we examined the associations of cannabis use and alcohol use with suicide using weighted multivariable logistic regression models to calculate adjusted odds ratios (aORs) and 95% confidence intervals (CIs). Bootstrap sampling was used to obtain valid estimates of aORs and 95% CIs (Lee et al. 2024). We fitted three models to generate the estimated aORs and 95% CIs: Model 1 was based on the complete NVDRS cases and NRS controls; Model 2 was based on NVDRS cases and NRS controls with 20 multiple imputations; and Model 3 was based on NVDRS cases and NSDUH controls with the data fusion approach and 20 multiple imputations.

## Results

There were considerable differences in demographic characteristics between cases (i.e., suicide deaths recorded in the 2013 NVDRS) and controls (i.e., respondents to the 2013 NSDUH) (Table 1). Specifically, cases were more likely than controls to be male (77.4% vs. 48.2, *p* < 0.001), be non-Hispanic White (84.9% vs. 65.5%, *p* < 0.001), and have received only a high school education or less (61.5% vs. 41.5%, *p* < 0.001). Age distribution, however, was similar between cases and controls (Table 1). As a select sample of drivers, participants in the 2013 NRS differed from both the NVDRS cases and the NSDUH controls and were disproportionately young adults aged 21–34 years (39.7% vs. 23.0% and 24.0%, *p* < 0.001), non-Hispanic Black (24.4% vs. 5.8% and 11.8%, *p* < 0.001), and attained education beyond high school (69.1% vs. 38.5% and 58.4%, *p* < 0.001) (Table 1).

**Table 1:** Demographic characteristics of study samples in three National data systems: 2013 National Violent Death Reporting System (NVDRS), 2013 National Roadside Survey of Alcohol and Drug Use by Drivers (NRS), and 2013 National Survey on Drug Use and Health (NSDUH)

Characteristics	NVDRS (*n* = 12,750) No. (%)	NRS (*n* = 11,314) No. (%)	NSDUH (*n* = 43,465) No. (%)
**Age**,** years**			
16–20	670 (5.3)	1,073 (11.7)	3,817 (8.8)
21–34	2,937 (23.0)	3,640 (39.7)	10,414 (24.0)
35–49	3,361 (26.4)	2,310 (25.2)	10,751 (24.7)
50–64	3,688 (28.9)	1,621 (17.7)	10,755 (24.7)
≥ 65	2,094 (16.4)	523 (5.7)	7,728 (17.8)
Missing	0	2,146	0
**Sex**			
Male	9,862 (77.4)	6,382 (58.3)	20,970 (48.2)
Female	2,873 (22.6)	4,566 (41.7)	22,495 (51.8)
Missing	15	365	0
**Race**			
White	10,831 (84.9)	4,952 (55.0)	28,458 (65.5)
Black	734 (5.8)	2,196 (24.4)	5,131 (11.8)
Hispanic	586 (4.6)	1,074 (11.9)	6,622 (15.2)
Other	599 (4.7)	776 (8.6)	3,254 (7.5)
Missing	0	2,316	0
**Education**			
Less than high school	1,350 (18.6)	715 (7.8)	5,616 (12.9)
High school graduate	3,105 (42.9)	2,115 (23.1)	12,441 (28.6)
Some college	1,124 (15.5)	3,222 (35.2)	11,365 (26.1)
College/Graduate	1,665 (23.0)	3,106 (33.9)	14,043 (32.3)
Missing	5,506	2,156	0

Over one quarter (28.7%) of the cases in NVDRS were tested for THC; of them, 16.9% were positive (Table 2). Of the NRS participants, 44.7% were tested for THC, with 9.4% being positive (Table 2). Blood alcohol testing results showed that 39.5% of the cases in NVDRS and 2.4% of the NRS participants had elevated BACs (Table 2). Results from multiply imputed data were consistent with those from actual testing data. The data fusion approach with multiple imputation estimated that of the NSDUH respondents, 7.2% were positive for THC and 2.6% were positive for alcohol (Table 2).

**Table 2:** Prevalence (%) and 95% confidence interval (CI) of cannabis use and alcohol use as indicated by positive blood tests according to actual testing data and multiply imputed data in the three National data systems: 2013 National Violent Death Reporting System (NVDRS), 2013 National Roadside Survey of Alcohol and Drug Use by Drivers (NRS), and 2013 National Survey on Drug Use and Health (NSDUH)

	NVDRS (*n* = 12,750) Prevalence (95% CI)	NRS (*n* = 11,314) Prevalence (95% CI)	NSDUH (*n* = 43,465) Prevalence (95% CI)
**Cannabis use**			
Actual testing data	16.90 (15.70,18.10)	9.37 (8.54, 10.20)	*
Multiply imputed data	17.10 (16.10, 17.90)	10.10 (9.45, 10.80)	7.20 (6.35, 7.90)
**Alcohol use**			
Actual testing data	39.50 (38.40, 40.70)	2.39(1.95, 2.82)	*
Multiply imputed data	39.20 (38.40, 40.00)	3.10 (2.90, 3.25)	2.63 (2.15, 3.35)

*Not available

Multivariable logistic regression modeling revealed that cannabis use and alcohol use were each significantly associated with increased odds of suicide (Table 3). The case-control analysis based on the data fusion approach yielded an aOR of 1.83 (95% CI: 1.17, 2.79) for the association between cannabis use and suicide and an aOR of 20.53 (95% CI: 11.83, 29.24) for the association between alcohol use and suicide (Table 3, Model 3). Results from the three models were similar. In all the models, significantly increased odds of suicide were found in males, non-Hispanic Whites,, and those with less education attainment (Table 3).

**Table 3:** Adjusted odds ratios (aORs) and 95% confidence intervals (CIs) of suicide from weighted multivariable logistic models: model 1 was based on the complete National Violent Death Reporting System (NVDRS) cases and the National Roadside Survey of Alcohol and Drug Use by Drivers (NRS) controls; model 2 was based on 20 multiply imputed NVDRS and NRS data; and model 3 used the data fusion technique, and 200 bootstrapped and 20 multiply imputed NVDRS and National Survey on Drug Use and Health (NSDUH) data

Variable	NVDRS + NRS		NVDRS + NSDUH
	Model 1 (*n* = 6,513)	Model 2 (*n* = 24,064)	Model 3 (*n* = 56,215)
	aOR (95% CI)	aOR (95% CI)	aOR (95% CI)
**Drug**			
**Cannabis (positive vs. negative)**	**1.81 (1.17**,** 2.79)**	**2.03 (1.59**,** 2.59)**	**1.83 (1.36**,** 2.31)**
Alcohol (positive vs. negative)	19.41 (13.94, 27.03)	19.58 (15.26, 25.13)	20.53 (11.83, 29.24)
**Age**,** years**			
35–49	1.00	1.00	1.00
16–20	0.26 (0.16, 0.40)	0.20 (0.15, 0.26)	0.58 (0.5, 0.65)
21–34	0.31 (0.25, 0.38)	0.32 (0.28, 0.35)	0.73 (0.63, 0.84)
50–64	0.61 (0.49, 0.76)	0.62 (0.54, 0.70)	0.91 (0.8, 1.02)
≥ 65	1.17 (0.88, 1.54)	1.74 (1.46, 2.07)	0.88 (0.78, 0.99)
**Sex**			
Female	1.00	1.00	1.00
Male	1.98 (1.70, 2.30)	2.07 (1.91, 2.25)	3.21 (2.95, 3.48)
**Race**			
White	1.00	1.00	1.00
Black	0.07 (0.03, 0.14)	0.14 (0.09, 0.24)	0.32 (0.27, 0.36)
Hispanic	0.64 (0.39, 1.06)	0.23 (0.15, 0.34)	0.19 (0.16, 0.22)
Others	0.46 (0.28, 0.74)	0.47 (0.33, 0.68)	0.58 (0.5, 0.66)
**Education**			
Less than high school	1.29 (0.98, 1.69)	1.50 (1.24, 1.81)	1.22 (1.09, 1.35)
High school graduate	1.00	1.00	1.00
Some college	0.35 (0.29, 0.42)	0.29 (0.25, 0.34)	0.44 (0.39, 0.49)
College graduate/Some graduate	0.46 (0.35, 0.60)	0.36 (0.29, 0.44)	0.5 (0.44, 0.55)

## Discussion

Our analysis of multiple national data systems indicates that cannabis use is associated with 83% increased odds of suicide. This finding is consistent with the Han et al. study (Han, et al., 2021), in which the prevalence of suicidality for cannabis users was significantly higher than for cannabis non-users among NSDUH respondents. The validity of our finding that there exists a robust association between cannabis use and increased suicide risk is further bolstered by the corroborative evidence that reaffirms several well-established risk factors for suicide, such as alcohol use, male sex, non-Hipanic White race, and less education attainment (Flensborg-Madsen et al. 2009; Borges et al. 2017; Peterson et al. 2020).

Our study contributes empirical evidence to the growing body of research linking cannabis use to increased suicide risk. Numerous studies have demonstrated a strong association between cannabis use and the development of psychotic disorders, such as schizophrenia (Volkow et al. 2014) and depression (Lev-Ran et al. 2014). These mental health issues can directly influence suicidal behavior, exacerbated by additional factors such as relationship problems, lower career achievement, and reduced life satisfaction. Furthermore, chronic cannabis use has been linked to cognitive and motor impairments, which may disrupt daily functioning and independence. For instance, impaired motor skills and slowed reaction time can affect driving ability, sometimes resulting in driving cessation—a loss of autonomy that has been associated with increased depression and suicide risk (Chihuri et al. 2016; Ko et al. 2021).

This study has several notable strengths. First, we used the case-control design and integrative data analysis techniques, including a novel data fusion approach to handling missing toxicological testing data. Selecting cases and controls from nationally representative samples enhances the generalizability of our findings compared to studies conducted in specific geographic regions or population groups. Additionally, by incorporating toxicological testing results instead of relying on self-reported data to determine cannabis use exposure, we minimized misclassification bias.

Several limitations should be taken into consideration when interpreting the study results. First, all toxicological testing data for NSDUH controls were imputed using prediction models trained on NRS data. This approach assumes that the relationship between self-reported and toxicology-confirmed use of cannabis and alcohol is consistent across populations. Differences in reporting behavior, such as underreporting in roadside survey settings, may limit calibration of the imputation model and underestimate uncertainty. Second, as NRS data cannot be disaggregated to state level, we could not restrict the analysis to the 17 states participating in the 2013 NVDRS. Differences in cannabis policy across states may therefore contribute to residual confounding. Also, our study may have limited applicability to certain racial and ethnic minorities due to inadequate representations in the study samples. Third, despite leveraging data fusion and multiple imputations to mitigate inaccuracies in self-reporting, selection bias might still be present due to the sampling methodology employed by NRS that targeted specific time windows on weekends. Fourth, the high prevalence of missing toxicological testing data on cannabis use from the NRS dataset may affect the accuracy of the multiply imputed data, as demonstrated in the discrepancies in the odds ratios derived from models before and after imputations using NRS data (Table 3). Fifth, our study results are susceptible to unmeasured confounding as some confounding factors, such as substance use disorder and history of depression and other mental health problems, are not accounted for due to the lack of data. Psychiatric conditions, such as major depression, schizophrenia, and anxiety disorder, have been recognized as important risk factors for suicide (Harris and Barrachlough 1997; Reutfors et al. 2009; Rivlin et al. 2010), and have been found to be positively associated with cannabis use (Bovasso 2001; Patton et al. 2002). Finally, this study does not assess the dose-response relationship between cannabis use and suicide because it is not feasible to multiply impute quantitative cannabis data.

## Conclusions

Results of this study indicate a positive association between cannabis use and suicide. Specifically, we found that cannabis use, as indicated by toxicological testing results, is associated with 83% increased odds of suicide with adjustment for demographic characteristics and alcohol use. If confirmed, the excess risk of suicide associated with cannabis use, along with other adverse health outcomes related to cannabis use, should be taken into consideration in the development and evaluation of cannabis policies.

## Data Availability

Data from the National Violent Death Reporting System are available from the Centers for Disease Control and Prevention. Data from the National Survey on Drug Use and Health are available from Substance Abuse and Mental Health Services Administration. Data from the National Roadside Survey of Alcohol and Drug Use by Drivers are available from the National Highway Traffic Safety Administration.

## Abbreviations

95% CI: 95% Confidence Interval
aOR: Adjusted Odds Ratio
BAC: Blood Alcohol Concentration
NRS: National Roadside Survey
NSDUH: National Survey on Drug Use and Health
NVDRS: National Violent Death Reporting System
OR: Odds Ratio
THC: Delta 9–tetrahydrocannabinol
